# Lurking in Ambush: A Case Report of Probable Creutzfeldt-Jakob Disease From Rural Kerala, India

**DOI:** 10.7759/cureus.95306

**Published:** 2025-10-24

**Authors:** Issac Georgy, Thomas V Pulickal, Calvin Jose, Alan J Kannemkuzhiyil, Avaroth Krishnadas

**Affiliations:** 1 Internal Medicine, Jayamatha Hospital, Kollam, IND; 2 Emergency Medicine, All India Institute of Medical Sciences, Bhubaneswar, Bhubaneswar, IND; 3 Internal Medicine, Good Shepherd Hospital, Wayanad, IND; 4 Family and Community Medicine, Good Shepherd Hospital, Wayanad, IND

**Keywords:** creutzfeldt-jakob disease, prions, rapidly progressive dementia, rural kerala, sporadic creutzfeldt-jakob disease

## Abstract

Creutzfeldt-Jakob disease (CJD) is a rare, rapidly progressive neurodegenerative disorder caused by prion proteins. This case report describes a probable instance of sporadic CJD in an elderly male from rural Kerala, India. The patient presented with rapidly evolving neurocognitive decline, myoclonus, and characteristic MRI and EEG findings. Despite limited resources, a probable diagnosis was established through clinical evaluation and supportive investigations. This case highlights the challenges of diagnosing rare neurological disorders in resource-constrained settings and underscores the importance of clinical vigilance in rural healthcare practice.

## Introduction

Creutzfeldt-Jakob disease (CJD) is the most common human prion disease, with a global incidence of approximately 0.5 to 1.5 cases per million population annually [1-3]. In India, the disease is significantly under-reported, with an estimated incidence of 0.085 per million population [4,5]. Limited awareness among healthcare professionals, the scarcity of specialized diagnostic facilities, and the absence of a robust national surveillance system contribute to this under-reporting [6,7]. The National Institute of Mental Health and Neurosciences (NIMHANS) in Bangalore recorded 105 CJD cases over the past 40 years in their National Registry [5]. The true prevalence remains elusive because of potential underdiagnosis and under-reporting [4-7]. This case report aims to contribute to the existing literature by detailing the clinical course, diagnostic challenges, and management of a patient with probable sporadic CJD in India.

## Case presentation

A 76-year-old male with hypertension, dyslipidemia, coronary artery disease, and a prior posterior circulation infarct presented with a one-month history of behavioral changes and increased forgetfulness, decreased personal care for two weeks, and an inability to recognize family members for one week. Caregivers reported that the patient had been cognitively and functionally normal until approximately four weeks prior to presentation. Over the subsequent week, he became apathetic and lost interest in routine activities. During the second week, they noted frequent forgetfulness regarding recent conversations and a tendency to misplace objects. By the end of the third week, his confusion had progressed, and during the last five to seven days, he became unable to recognize familiar people. Intermittent myoclonic jerks, often precipitated by startle, were first observed about two weeks earlier and had gradually increased in frequency. There was no history of trauma, vomiting, or fever at presentation. 

On examination, he was disoriented to time, place, and person. Speech fluency was reduced, comprehension was preserved, nominal aphasia was present, and both reading and writing abilities were impaired, with repetition similarly affected. Memory was impaired across the immediate, recent, and remote domains. Apraxia was evident, and startle-induced myoclonus was observed. The motor examination revealed hypertonic tone and showed muscle power of 3/5 in all four limbs. Deep-tendon reflexes were brisk, and plantar responses were bilaterally extensor. Cerebellar signs were present, and gait could not be assessed because of severe weakness and postural instability. Visual acuity was reduced, detailed cranial nerve function couldn’t be assessed due to the apraxia, no meningeal signs. Peripheral oxygen saturation ranged between 94-98% on room air, blood pressure fluctuated from 130/90 mmHg to 160/100 mmHg, and heart rate varied from 90 to 112 beats per minute.

Initial investigations, including a complete blood count (CBC), basal metabolic panel (BMP), and liver function test (LFT), showed a normal picture, except for a glucose level of 212 mg/dL. The lipid profile was deranged. HIV, HBsAg, and VDRL tests came out to be non-reactive (Table 1).

**Table 1:** Initial investigations including CBC, BMP, LFT, lipid profile, and infection screening CBC: complete blood count, BMP: basal metabolic panel, LFT: liver function test

Investigation	Patient's level	Normal reference range
Random blood glucose	212 mg/dl	<140 mg/dl
CBC (complete blood count)		
Hemoglobin	16 g/dL	13.5–17.5 g/dL (men)
Total WBC count	10,500 /mm³	4,000–11,000 /mm³
Neutrophils	56%	40–70%
Lymphocytes	34%	20–40%
Monocytes	5%	2–8%
Eosinophils	4%	1–4%
Basophils	1%	0–1%
Platelet count	360,000 /mm³	150,000–450,000/mm³
RBC count	5.7 million/µL	4.7–6.1 million/µL (men)
Hematocrit	47%	41–53% (men)
MCV	92 fL	80–100 fL
MCH	32 pg	27–33 pg
MCHC	34 g/dL	32–36 g/dL
BMP (basic metabolic panel)		
Sodium (Na⁺)	135 mEq/L	135–145 mEq/L
Potassium (K⁺)	4.1 mEq/L	3.5–5.0 mEq/L
Chloride (Cl⁻)	104 mEq/L	98–106 mEq/L
Bicarbonate (HCO₃⁻)	26 mEq/L	22–28 mEq/L
Blood urea nitrogen (BUN)	8 mg/dL	7–20 mg/dL
Creatinine	0.8 mg/dL	0.6–1.2 mg/dL
Glucose (Fasting)	198 mg/dL	70–99 mg/dL
Calcium	10.1 mg/dL	8.5–10.5 mg/dL
Liver function tests (LFTs)		
AST (SGOT)	45 U/L	10–40 U/L
ALT (SGPT)	54 U/L	7–56 U/L
ALP (alkaline phosphatase)	140 U/L	44–147 U/L
Total bilirubin	1.1 mg/dL	0.3–1.2 mg/dL
Direct bilirubin	0.2 mg/dL	<0.3 mg/dL
Albumin	3.6 g/dL	3.5–5.0 g/dL
Total protein	6.4 3.6 g/dL	6.0–8.3 g/dL
GGT	46 U/L	9–48 U/L
Prothrombin time (PT)	12.5 sec	11–13.5 sec
INR	0.9	0.8–1.1
Lipid profile		
Total cholesterol	256 mg/dL	<200 mg/dL
LDL cholesterol	170 mg/dL	<100 mg/dL
HDL cholesterol	35 mg/dL	>40 mg/dL (men)
Triglycerides	202 mg/dL	<150 mg/dL
VLDL cholesterol	36 mg/dL	5–40 mg/dL
TC/HDL ratio	7.3	<5 (ideal <3.5) Ratio
Non-HDL cholesterol	155 mg/dL	<130 mg/dL
HIV 1 & 2 ELISA	Non-reactive (negative)	Non-reactive (negative)
HBsAg	Non-reactive (negative)	Non-reactive (negative)
VDRL	Non-reactive (negative)	Non-reactive (negative)

**Figure 1:**
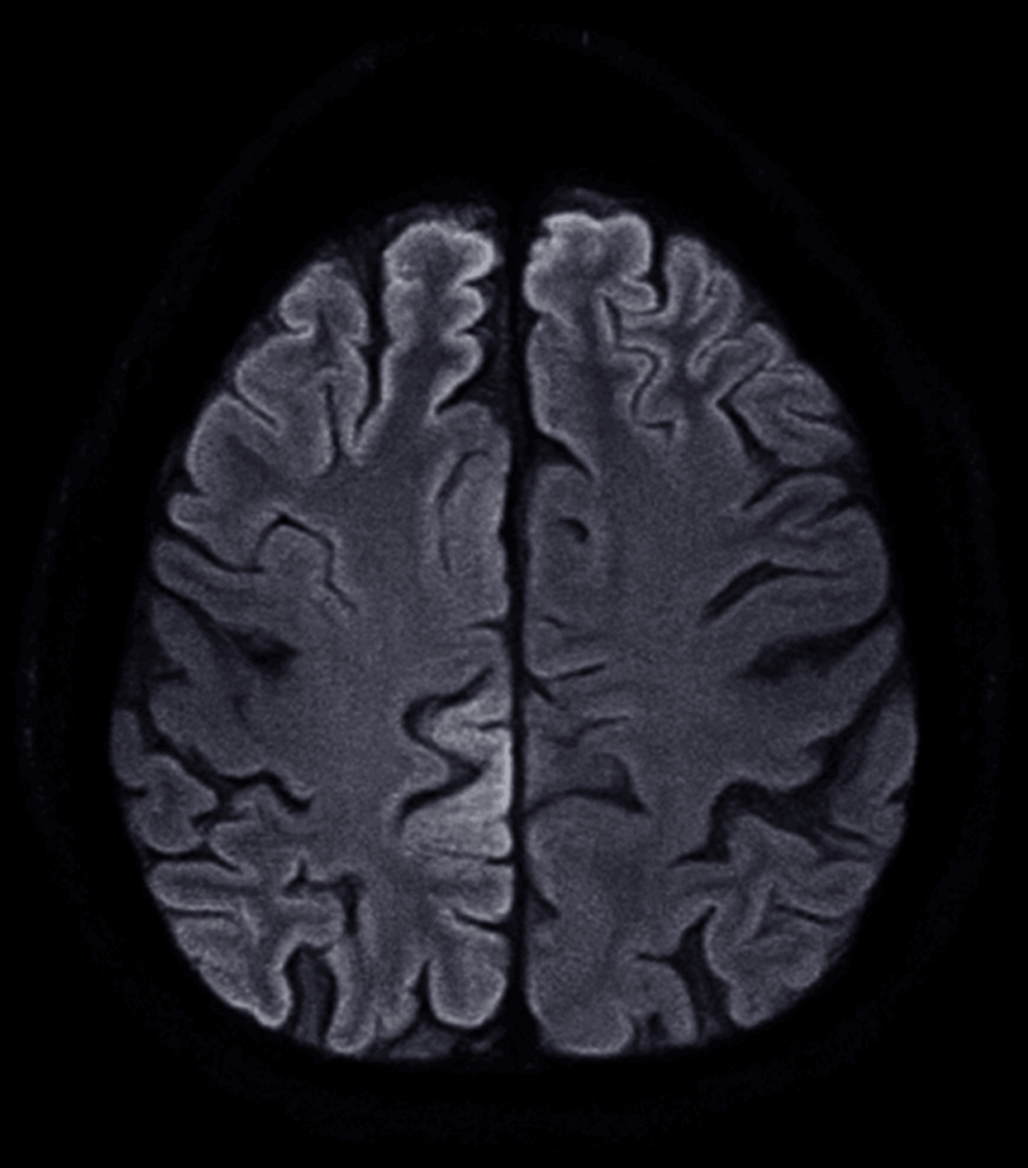
MRI brain ​​​​​​​diffusion-weighted imaging (DWI) and FLAIR sequence shows cortical FLAIR hyperintensities with diffusion restriction in the bilateral frontal and parietal lobes, demonstrating the characteristic "ribboning appearance." FLAIR: fluid-attenuated inversion recovery

**Figure 2:**
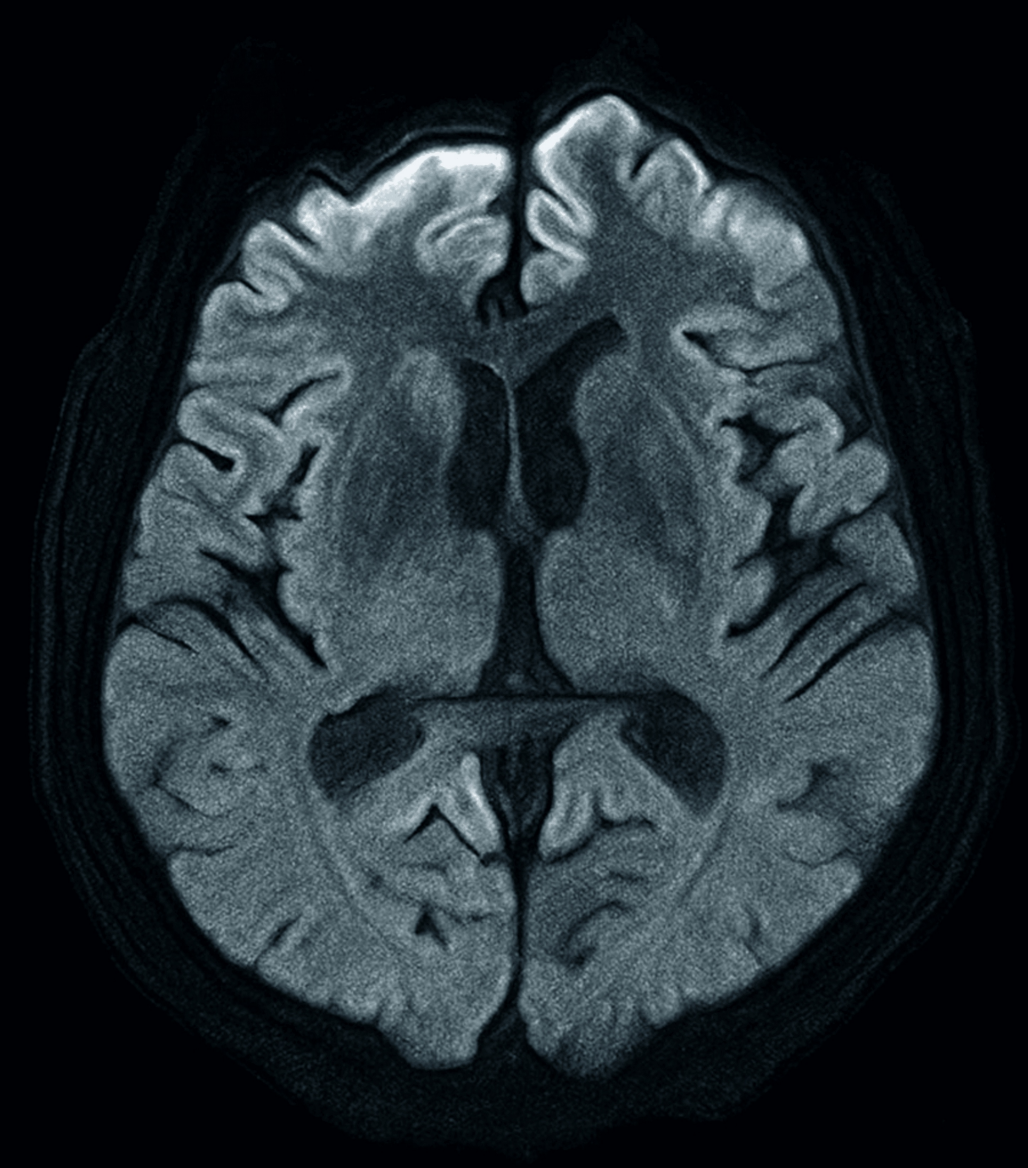
MRI brain ​​​​​​​diffusion-weighted imaging (DWI) and FLAIR sequence shows diffusion restriction in the head of the caudate nucleus, along with the characteristic cortical “ribboning appearance.” FLAIR: fluid-attenuated inversion recovery

**Figure 3:**
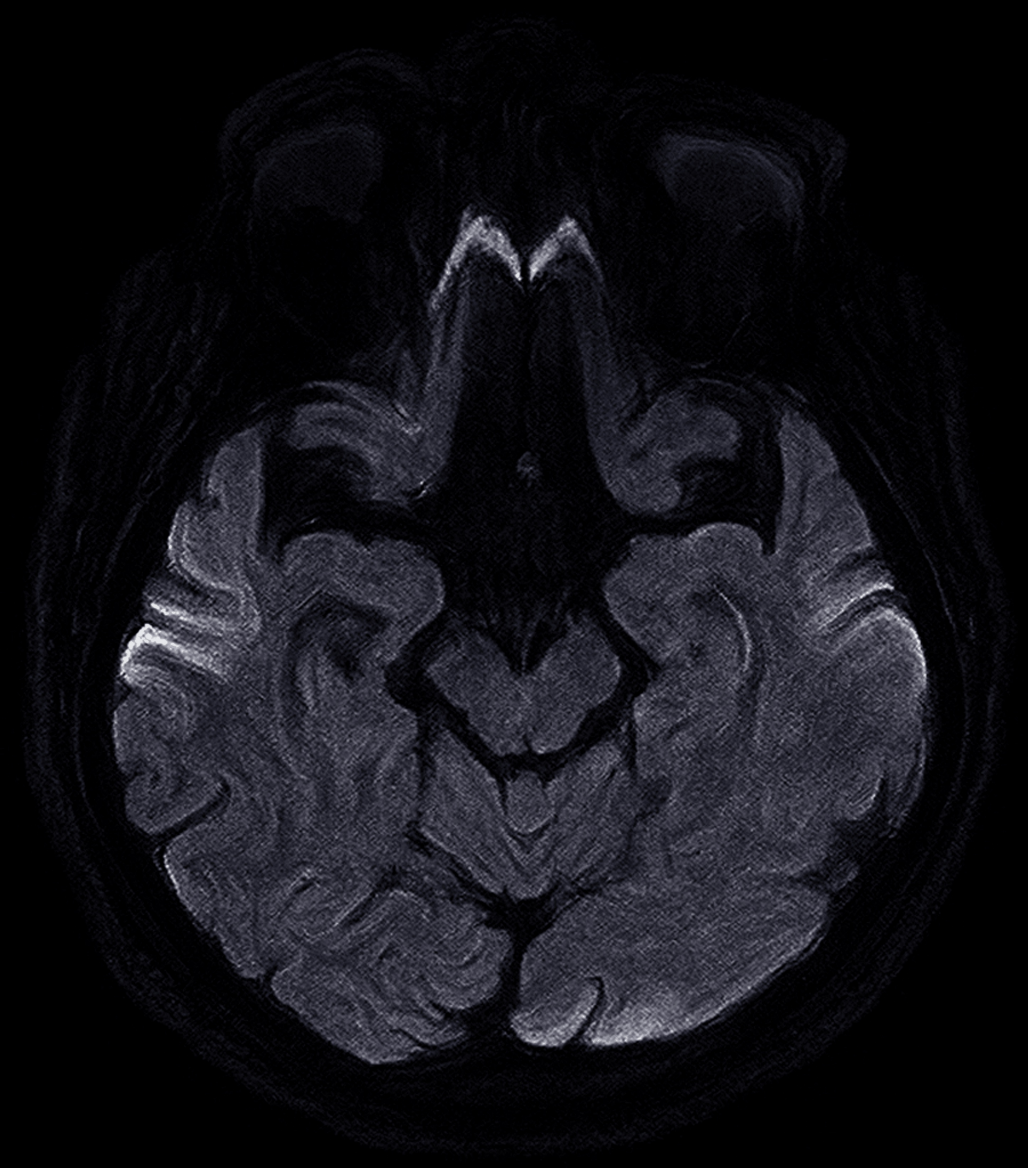
MRI brain ​​​​​​​diffusion-weighted imaging (DWI) and FLAIR sequence shows cortical FLAIR hyperintensities and diffusion restriction involving the bilateral parietal lobes. FLAIR: fluid-attenuated inversion recovery

EKG showed a normal sinus rhythm and movement artifacts in the limb leads due to the myoclonus. MRI brain screening demonstrated cortical FLAIR hyperintensities with diffusion restriction in the bilateral parietal and frontal regions, subtle diffusion restriction in the head of the caudate nucleus, and mild small-vessel ischemic changes; the overall impression was suggestive of a prion disease, most likely CJD [8-10] (Figures 1-3).

Electroencephalography showed a severe degree of global cerebral dysfunction consistent with diffuse encephalopathy, without obvious signs of seizures [11]. A routine CSF analysis showed a normal picture (Table 2).

**Table 2:** Routine cerebrospinal fluid (CSF) analysis

Investigation	Patient's level	Normal reference range
Cerebrospinal fluid (CSF) analysis		
Appearance	Clear	Clear, colorless
Opening pressure	160 mm H₂O	90–180 mm H₂O
WBCs	4 cells/mm³	0–5 cells/mm³ (mostly lymphocytes)
RBCs	2 cells/mm³	0 cells/mm³
Protein	50mg/dL	15–45 mg/dL
Glucose	60mg/dL	45–80 mg/dL or ≥60% of serum glucose
Chloride	130 mEq/L	118–132 mEq/L
Culture	No growth	No growth
Gram stain	No organisms seen	No organisms seen
Oligoclonal bands	Negative	Negative

Due to the high suspicion from the clinical course, examination, and radiological findings, a specific CSF analysis on the 14-3-3 protein gamma level was sent to a specialist lab [12]. Although the test was costly, the family consented to proceed, and arrangements were made to transfer the patient to the designated higher-level care center. The results revealed a 14-3-3 protein gamma level of 146,396 AU/mL, well above the 100,000 AU/mL threshold that indicates a very high risk for CJD (Table 3).

The more advanced tests, like RT-QuIC (real-time quaking-induced conversion), CSF Tau protein, and S-100, could not be performed due to limited availability and financial constraints. RT‑QuIC has higher specificity than 14-3-3 protein analysis on CSF and is now incorporated into updated criteria.

Despite supportive management, the patient’s condition deteriorated. The combination of rapidly progressive dementia, myoclonus, the characteristic MRI and EEG findings, and the highly elevated CSF 14-3-3 protein level fulfilled the CDC diagnostic criteria for probable sporadic CJD [13]. Home-based palliative care was provided at the family’s request, including nutrition management, dysphagia precautions, aspiration prevention, and discussions on advance directives to improve clinical utility. He died six months later, due to aspiration pneumonia, a course consistent with the expected prognosis of CJD [14]. A brain biopsy could not be performed due to cultural constraints. Therefore, the case remains classified as probable CJD rather than a definitive, biopsy-confirmed diagnosis.

## Discussion

CJD is a rare, fatal neurodegenerative disorder characterized by rapidly progressive dementia, myoclonus, and a range of neurological symptoms [6,9]. It belongs to the transmissible spongiform encephalopathies and is caused by misfolded prion proteins that lead to neuronal loss and spongiform changes in the brain [12]. The disease can be sporadic, familial, iatrogenic, or variant, with sporadic cases accounting for roughly 85-95% of the global total. Globally, the incidence of CJD is estimated at 0.5 to 1.5 cases per million population annually [13]

In India, epidemiological data are limited; the National Institute of Mental Health and Neurosciences (NIMHANS) recorded 69 cases between 1968 and 1997 [4-6], a North Indian series reported 10 cases between 1990 and 1998 [5], and an Eastern Indian study identified eight probable cases over three years [7]. The current registry of NIMHANS has 105 reported cases of CJD over the past 40 years [6]. Overall prevalence remains uncertain because diagnostic challenges and inadequate surveillance obscure the true burden [4-7].

CJD has a prolonged incubation period ranging from 15 months to over 30 years. It typically presents between the ages of 55 and 75, with a mean age of onset around 60 years [10,13]. Once symptoms appear, the disease is rapidly progressive and usually proves fatal within one year. Common manifestations include rapidly progressive cognitive decline affecting memory, judgment, and higher thinking, startle-induced myoclonus, and other neurological signs such as ataxia, visual disturbances, and pyramidal or extrapyramidal involvement, together with behavioural changes including personality alterations, depression, and psychosis [13].

An MRI brain diffusion-weighted imaging (DWI) and FLAIR sequences show a classical "cortical ribboning" appearance due to the cortical hyperintensities and restricted diffusion with widespread atrophy. Hyperintensities can also be seen in the caudate nucleus and putamen. Classical signs like Pulvinar/hockey‑stick signs are more typical of variant CJD [14]. EEG shows early diffuse slowing activity, middle sharp wave complexes, and non-reactive coma traces towards the later stages [15].

A 14-3-3 protein analysis in the CSF sample was shown to be highly sensitive and specific for the diagnosis of CJD [16]. A newer assay RT-QuIC (real-time quaking-induced conversion) was described in 2010 with higher sensitivity and specificity than the 14-3-3 protein analysis, even to detect a small amount of prion proteins [17]. A latest modification to it called Nano-QuIC has been developed by the Minnesota Center for Prion Research and Outreach(MNPRO), which reduces the detection time to just four hours and increases the sensitivity to a factor of 10 [18].

In India, the diagnosis of CJD is hindered by limited clinical awareness; restricted access to advanced diagnostic tools such as MRI, EEG, CSF 14-3-3 protein assays, and RT-QuIC; and significant underreporting factors that collectively impede accurate epidemiological surveillance.

The prions had been lurking for 20-30 years, silently awaiting the moment to ambush and rapidly deteriorate the individual, hence the title “Lurking in Ambush” for this case.

## Conclusions

This case illustrates the critical need for greater clinical vigilance and improved diagnostic infrastructure for CJD in India. Early recognition and accurate diagnosis support better patient care and informed family counselling, yet these goals depend on enhanced awareness among clinicians and a robust surveillance framework. Further research and policy action are required to close persistent gaps in the identification and reporting of CJD in the Indian context.
